# Microsatellite instability and Beta2-Microglobulin mutations as prognostic markers in colon cancer: results of the FOGT-4 trial

**DOI:** 10.1038/bjc.2012.53

**Published:** 2012-02-21

**Authors:** A Tikidzhieva, A Benner, S Michel, A Formentini, K-H Link, W Dippold, M von Knebel Doeberitz, M Kornmann, M Kloor

**Affiliations:** 1 Department of Applied Tumor Biology, Institute of Pathology, University Hospital Heidelberg, Im Neuenheimer Feld 220/221, Heidelberg 69120, Germany; 2 Clinical Cooperation Unit Applied Tumor Biology, German Cancer Research Center (DKFZ), Im Neuenheimer Feld 220/221, Heidelberg 69120, Germany; 3 Department of Biostatistics, German Cancer Research Center (DKFZ), Im Neuenheimer Feld 280, Heidelberg 69120, Germany; 4 Department of General, Visceral, and Transplantation Surgery, University of Ulm Steinhövelstr. 9, Ulm 89075, Germany; 5 FOGT-Study Group, Asklepios Paulinen Klinik, Geisenheimer Strasse 10, Wiesbaden 65197, Germany; 6 Department of Internal Medicine, St. Vincenz-and-Elisabeth Hospital Mainz, An der Goldgrube 11, Mainz 55131, Germany

**Keywords:** adjuvant chemotherapy, beta2-Microglobulin, colon cancer, microsatellite instability, prognostic marker

## Abstract

**Background:**

High-level microsatellite instability (MSI-H) has been reported as a prognostic marker in colon cancer. We here analysed the prognostic significance of MSI and mutations of the *Beta2-Microglobulin* (*B2M*) gene, which occur in about 30% of MSI-H colon cancer, in the cohort of the prospective FOGT-4 (Forschungsruppe Onkologie Gastrointestinale Tumoren, FOGT) trial.

**Methods:**

Microsatellite instability status was determined using standard protocols (NCI/ICG-HNPCC panel and CAT25) in 223 colon cancer lesions. *Beta2-Microglobulin* mutation status was evaluated by exon-wise sequencing in all MSI-H lesions.

**Results:**

Patients with MSI-H (*n*=34) colon cancer presented with a significantly lower risk of relapse after 12 months of follow-up compared with MSS (*n*=189) colon cancer patients (5 year time to relapse: MSI-H 0.82 *vs* MSS 0.66, *P*=0.03). No significant difference in overall survival was detected. *Beta2-Microglobulin* mutations were identified in 10 (29.4%) out of 34 MSI-H colon cancers and were associated with a complete absence of disease relapse or tumour-related death events (*P*=0.09).

**Conclusion:**

The risk of late disease relapse was significantly lower in patients with MSI-H compared with MSS colon cancer. Moreover, *B2M* mutations may contribute to the favourable outcome of MSI-H colon cancer patients and should therefore be evaluated as a potential prognostic marker in future clinical trials.

Colorectal cancer is a pathogenetically heterogeneous disease. The majority of colorectal cancers display chromosomal instability and follow the classical adenoma-carcinoma sequence (Vogelstein *et al*, 1988; Lengauer *et al*, 1997). About 15% of colorectal cancers occur as a consequence of defects in the DNA mismatch repair (MMR) system. These cancers are characterised by high-level microsatellite instability (MSI-H), that is, the accumulation of multiple insertion/deletion mutations at short repetitive sequence stretches in the genome. High-level MSI is observed more frequently in colon cancers that are located proximally to the splenic flexure (Gryfe *et al*, 2000). High-level MSI colon cancers are commonly poorly differentiated, often presenting with a mucinous or mixed histology (Ward *et al*, 2001; Shia *et al*, 2003). Patients with MSI-H colon cancers rarely develop distant metastasis and have a longer overall survival (OS) than stage-matched MSS colon cancer patients (Gryfe *et al*, 2000; Buckowitz *et al*, 2005; Popat *et al*, 2005).

Microsatellite instability status has been discussed as a potential predictor of therapy outcome after adjuvant treatment with 5-fluorouracil (5-FU), because MSI-H colon cancer patients did not benefit from 5-FU-based chemotherapy in an adjuvant setting (Ribic *et al*, 2003). The rate of 5-year disease-free survival in MSI-H patients not receiving adjuvant chemotherapy tended to be higher (88%) than in MSI-H patients receiving adjuvant chemotherapy (71% Ribic *et al*, 2003). Although there is no conclusive evidence for a beneficial effect of irinotecan (CPT-11) in adjuvant treatment of colon cancer (Van Cutsem *et al*, 2009; Ychou *et al*, 2009; Papadimitriou *et al*, 2011; Sargent *et al*, 2011), an association between MSI-H status and benefit from irinotecan-based chemotherapy could be demonstrated by Fallik *et al* (2003). A similar trend was later observed in a randomised prospective trial using irinotecan (Bertagnolli *et al*, 2009). Accordingly, irinotecan might represent an option for adjuvant therapy specifically in MSI-H colon cancer patients (Gasparini *et al*, 2010).

More recently, explorative retrospective studies have revealed a novel marker that might allow for a prognostic subclassification of MSI-H colon cancers in two groups with a different metastatic behaviour: *Beta2-Microglobulin* (*B2M*) mutations were detected at a frequency of ∼40% in stage III MSI-H colon cancer lesions, but were completely absent in stage IV MSI-H colon cancers (Kloor *et al*, 2007). *Beta2-Microglobulin* mutations are localised at coding microsatellite stretches and result from MMR deficiency, thus occurring almost exclusively in MSI-H tumours (Bicknell *et al*, 1996; Kloor *et al*, 2005). *Beta2-Microglobulin* mutations lead to a complete loss of HLA class I-mediated antigen presentation and represent the most important mechanism of immune evasion in MSI-H colon cancers (Bicknell *et al*, 1996; Kloor *et al*, 2005). The prognostic significance of *B2M* mutations had not yet been examined in a prospective setting.

The controlled, prospective FOGT-4 phase III trial had been initiated to improve outcome of patients with locally advanced colon cancer and compared the efficacy of 5-FU/FA (folinic acid) with the combination of 5-FU/FA and irinotecan for adjuvant chemotherapy of colon cancer. The aim of the present analysis is to investigate the influence of MSI-H and mutations of the *B2M* gene on patients' outcome in the FOGT-4 cohort.

## Materials and Methods

### Trial design

The German ‘Research Group Oncology of Gastrointestinal Tumors’ (Forschungsruppe Onkologie Gastrointestinale Tumoren, FOGT) designed the prospective randomised FOGT-4 trial to optimise adjuvant treatment of locally advanced colon cancer, conforming to GCP/ICH rules, respecting the Helsinki Declaration (1989) Principles, and having been approved by the Ethics Committee of the University of Ulm (#72/2001). An independent study monitor supervised the trial. The aim of the study was to increase OS in locally advanced colon cancer by combining standard adjuvant 5-FU/FA chemotherapy with irinotecan. Primary end point was OS, and secondary end points were recurrence-free survival, toxicity, quality of life and determination of predictive and prognostic makers for treatment as previously reported (Staib *et al*, 2010). A total of 600 patients were planned to be included. Owing to a dramatically decreasing frequency of enrolment after initial publication of three other trials, reporting no beneficial results, about the efficacy of the addition of irinotecan to 5-FU/FA in adjuvant treatment of colon cancer (Sargent *et al*, 2009; Van Cutsem *et al*, 2009; Ychou *et al*, 2009), our study group decided to stop the FOGT-4 trial after inclusion of 281 patients.

### Patient eligibility criteria

Eligible were patients (⩾18 years) with a potentially curative en-bloc resection (R_0_) of an adenocarcinoma of the colon displaying a pathologic UICC stage IIb (pT4pN0M0) or III (pT1-4pNposM0), according to the 6th version of the UICC/TNM classification (Link *et al*, 2005; Staib *et al*, 2010). Ineligibility criteria were as published (Link *et al*, 2005) and included a history of secondary cancer, any systemic chemotherapy, pregnant or nursing women, a known allergy towards irinotecanhydroclorid or of any ingredients of Campto, other severe medical, laboratory and social conditions not allowing chemotherapy and follow-up.

### Stratification and randomisation procedures

Patients were randomised following stratification according to the centre, pathological T classification and pathological lymph node status to receive postoperative treatment with 5-FU/FA or with 5-FU/FA and irinotecan (FOLFIRI).

### Chemotherapy

Standard 5-FU/FA was administered as described (Link *et al*, 2005). Folinic acid (200 mg m^−2^ body surface area) was administered as short intravenously (i.v.) infusion for 10 min, followed by systemic 5-FU (450 mg m^−2^) for 120 min. In case of FOLFIRI, irinotecan (Campto, 80 mg m^−2^) was administered i.v. for 60 min after subcutaneous pretreatment with 0.25 mg atropin, followed by a 120-min infusion of FA (500 mg m^−2^) and a 24-h infusion of 5-FU (2.000 mg m^−2^). Start of chemotherapy was usually scheduled for day 14 to day 28 after surgery. 5-Fluorouracil/FA treatment was administered as a loading course on days 1–5, followed by a 3-week break and thereafter administered once weekly for 6 months. FOLFIRI treatment was administered via a port system starting on day 1 once weekly for 6 weeks followed by a 2-week break and repeated 4 times. In patients >70 years of age, it was allowed to start the treatment at a reduced dosage of 80%, especially for FOLFIRI.

### Patient data

Primary patient data as well as follow-up data were obtained from the central study centre (European Trial and Medical Support, Altdorf, Germany). They included date of birth, gender, date of operation, pathological staging and grading, type of chemotherapy, location(s) and date of first recurrence, date of death, reason of death and last observation date for patients being alive. Staging and follow-up procedures were carried out every 4 months for 2 years, followed by 6-month intervals for 3 years and an optimal annual follow-up for another 5 years as described previously (Link *et al*, 2005). For staging, the sixth edition of the UICC/AJCC TNM system (2002) was applied.

### Tissue retrieval and DNA isolation

Paraffin-embedded primary tumour tissue from patients was prospectively collected at the study centre in Ulm. Two 5-*μ*m sections were prepared from each paraffin block. Genomic DNA was extracted after manual microdissection using the DNeasy tissue kit (Qiagen, Hilden, Germany) following the manufacturer's recommendation. Overall, molecular evaluation of primary tumour tissue was successful in 223 (82.9%) of the 269 evaluable patients of the FOGT-4 trial.

### MSI typing

Microsatellite instability typing was performed using the marker panel CAT25, BAT25 and BAT26, as described previously (Findeisen *et al*, 2005). High-level MSI was scored if at least two out of three markers showed MSI.

### *Beta2-Microglobulin* mutation analysis

For *B2M* mutation analysis, exon-wise sequencing was performed as described previously (Kloor *et al*, 2007). The following primers were used for amplification and sequencing reaction: forward primer 5′-GGCATTCCTGAAGCTGACA-3′ and reverse primer 5′-AGAGCGGGAGAGGAAGGAC-3′ for exon 1, forward primer 5′-ACCCTGGCAATATTAATGTGTC-3′ and reverse primer 5′-TACACAACTTTCAGCAGTTAC-3′ for exon 2.

### Statistical analysis

Pairwise comparisons of continuous data were performed using the Wilcoxon rank-sum test. For binary data, Fisher's exact test was used, whereas for ordinal categorical data the Cochran-Armitage trend test was applied. Overall survival was defined as time from surgery to date of death of any cause. For time to relapse (TTR), defined as the time from surgery until objective tumour relapse, deaths before relapse were censored at the time point of last follow-up examination. Survival distributions were estimated by the method of Kaplan and Meier, and compared using the log-rank test. To account for possibly crossing hazards, we used the two-stage procedure as proposed in Qiu and Sheng (2008). Median follow-up time was estimated using the reverse Kaplan–Meier method (Schemper and Smith, 1996). To test the association of MSI status and *B2M* mutation with OS and time to relapse, multivariable Cox regression models were used. Additional covariates included in the models were age, gender, lymph node involvement and treatment. The proportional hazards assumption was tested as proposed in Grambsch and Therneau (1994). In addition to standard Cox models, we used the approach proposed in Scheike and Martinussen (2004) to include time-varying covariate effects into the Cox model.

## Results

### Patient data and microsatellite instability status

The FOGT-4 cohort was retrospectively analysed for MSI, and *B2M* mutation status as potential prognostic or predictive markers in colon cancer. In total, 281 patients were initially assessed for eligibility, 12 patients were excluded either due to withdrawn consent or other reasons (e.g., detection of metastases during staging, refusal of chemotherapy), so that 269 patients finally remained for analysis. Molecular characterisation of MSI status was feasible in 223 colon cancer lesions defining the evaluable sample population (ESP). No significant differences with respect to clinical characteristics could be found between patients being evaluable for MSI and patients not being evaluable for MSI. Of 223 tumour samples tested for MSI, 34 (15.2%) demonstrated the MSI-H phenotype. These MSI-H tumours were further analysed for *B2M* mutation status.

Median age was 66 years for MSI-H colon cancer patients, and 64 years for MSS colon cancer patients. There were no significant differences between the groups with regard to age, gender, tumour stage, lymph node involvement or treatment.

Clinical characteristics in relation to ESP, MSI status and *B2M* mutation status are summarised in Table 1.

### Chemotherapy and survival

**Figure 1:**
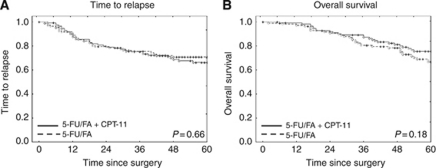
Kaplan–Meier estimates of OS and TTR in patients of FOGT-4 cohort according to therapy. (**A**) Time to relapse. No significant difference of TTR was observed between the 5-FU/FA and the 5-FU/FA + CPT-11 (FOLFIRI) groups. (**B**) Overall survival. Overall survival tended to be better in the 5-FU/FA + CPT-11 (FOLFIRI) group compared to the 5-FU/FA group, but differences were not significant.

No significant differences of survival curves with respect to TTR or OS were found between patients who were treated with adjuvant 5-FU/FA chemotherapy and those who were treated with 5-FU/FA in combination with irinotecan (5-FU/FA + CPT-11, FOLFIRI; Figure 1).

### Microsatellite instability status and survival

**Figure 2:**
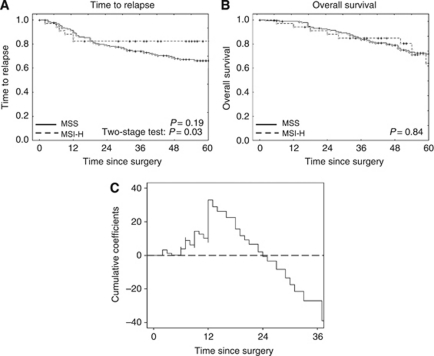
(**A** and **B**) Kaplan–Meier estimates of OS and TTR in patients of FOGT-4 cohort according to MSI status of tumour. (**A**) Time to relapse. No significant difference of TTR was observed between the MSI-H and the MSS groups when analysed using the log-rank test. Accounting for the crossing hazards, a two-stage test was applied, demonstrating a significantly different outcome of MSI-H compared with MSS colon cancer patients with improved prognosis after a period of 12 months. (**B**) Overall survival. No significant difference of overall survival was observed between the MSI-H and the MSS colon cancer groups. (**C**) Cumulative coefficient for MSI-H (log-Hazard over time). A value of zero (dashed line) indicates equal cumulative risks of disease relapse in the MSI-H and MSS groups. Abbreviation: MSS=microsatellite stable.

No significant difference in OS was detected between MSI-H and MSS colon cancer patients (Figure 2B), irrespective of the chemotherapeutic regimen applied (data not shown). Time to relapse revealed a significantly different outcome of MSI-H compared with MSS colon cancer patients with a lower risk for MSI-H patients, particularly for the period later than 12 months after surgery, resulting in 5-year TTR estimates of 0.82 for MSI-H *vs* 0.66 for MSS patients (Qiu and Sheng's two-stage test *P*=0.03). Relapse events in the MSI-H colon cancer subgroup were restricted to the first 12 months after surgical tumour resection (Figure 2A). After adjusting for other covariates, the time-dependent effect of MSI remained statistically significant (Table 2; Figure 2C). Separate analysis of UICC stage III colon cancer patients showed similar results (data not shown), whereas separate analysis of UICC stage II patients was not feasible due to the limited sample size (*n*=32).

### *Beta2-Microglobulin* mutation status and survival in MSI-H patients

**Figure 3:**
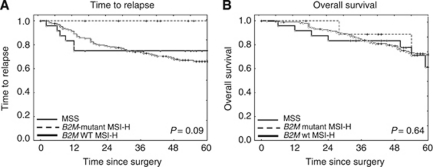
Kaplan–Meier estimates of survival in dependence of *B2M* mutation status. (**A**) Time to relapse. No relapse events were observed in *B2M*-mutant MSI-H colon cancer patients, whereas relapse events occurred in *B2M* wild-type (WT) MSI-H colon cancer and MSS colon cancer patients. (**B**) Overall survival. No significant difference of overall survival was observed between the *B2M*-mutant and *B2M* WT MSI-H colon cancer groups. Abbreviation: MSS=microsatellite stable.

In the subgroup of MSI-H colon cancer patients, *B2M* mutation status was determined to evaluate potential associations of *B2M* mutations with prognosis in MSI-H colon cancer. Truncating *B2M* mutations were identified in 10 (29.4%) out of 34 MSI-H colon cancers. No relapse events were observed in patients with *B2M*-mutant MSI-H colon cancer (Figure 3A), whereas relapse events occurred in 6 (25.0%) out of 24 *B2M* wild-type MSI-H colon cancer patients within the first 12 months after surgery (log-rank test *P*=0.09). A similar trend towards improved outcome of patients with *B2M*-mutant tumours was observed when analysing tumour-related deaths, as no tumour-related deaths were observed in *B2M*-mutant colon cancer patients (data not shown); however, differences in OS were not statistically significant (log-rank test *P*=0.64).

## Discussion

This study aimed at the evaluation of MSI and *B2M* mutation status as potential prognostic or predictive markers in patients with colon cancer, eligible for adjuvant chemotherapy enrolled in the controlled prospective FOGT-4 chemotherapy trial.

The FOGT-4 trial did not reveal any significant differences in OS or TTR in dependence of the chemotherapy regimen (5-FU/FA *vs* FOLFIRI). This is in line with previous studies suggesting that irinotecan-based chemotherapy does not have a beneficial effect on colon cancer patients' survival in an adjuvant setting (Sargent *et al*, 2009; Van Cutsem *et al*, 2009; Ychou *et al*, 2009). Irinotecan-based chemotherapy has been discussed as a potential therapeutic option in MSI-H colon cancer patients, because a particular responsiveness towards irinotecan-based chemotherapy had been observed in MSI-H colon cancers (Fallik *et al*, 2003). A favourable effect of irinotecan in MSI-H colon cancer patients has recently been suggested by the results of a clinical trial that compared 5-FU/leucovorin with 5-FU/leucovorin/irinotecan as an adjuvant chemotherapy in stage III colon cancer patients (Bertagnolli *et al*, 2009). The analysis of MSI-H-specific effects on chemotherapy response in this study is difficult, because its power is limited due to the fact that the FOGT-4 trial was stopped after inclusion of 281 patients, of which 223 were available for molecular analysis defining the ESP set. In particular, a separate analysis of the MSI-H CRC patients' subgroup for chemotherapy response was not feasible due to the limited number of patients and relapse or death events (data not shown). Thus, this study is underpowered for the evaluation of irinotecan-based chemotherapy in MSI-H CRC patients.

The MSI-H phenotype was detected in 15.2% of analysed colon cancer lesions from the FOGT-4 study cohort. This is in line with MSI-H frequencies reported in the literature for unselected cohorts of colon cancer patients (Haydon and Jass, 2002; Ogino *et al*, 2009; Vilar and Gruber, 2010). Our study revealed no significant differences between MSI-H and MSS colon cancer patients concerning OS. However, significant differences between the MSI-H and MSS groups were observed with regard to TTR. The risk of disease relapse within the first 12 months after surgery was elevated among MSI-H compared with MSS colon cancer patients, whereas this trend reversed starting in years 2 and 3 of follow-up, with an equal cumulative risk of disease relapse ∼2 years after surgery. A similar trend towards an initially higher rate of disease relapses in MSI-H compared with MSS colon cancer patients can be seen in survival curves reported by Bertagnolli *et al* (2009). Notably, in our study all relapse events affecting patients of the MSI-H colon cancer group occurred within the first year after surgery, and no relapse events were observed at a later time point. This is in contrast to the MSS colon cancer group, where the distribution of hazards rather appeared to be uniform over the whole span of 5 years. Our results indicate that the mechanism leading to disease relapse in colon cancer patients depends on the MSI status of the tumour. Further studies on the causes of early disease recurrence in MSI-H colon cancer patients are warranted, contributing to a better understanding and potentially adapted management recommendations for surgery and/or post-operative treatment of these patients.

Five-year OS was about 70% for the MSI-H and MSS colon cancer patients in this study. This almost exactly matches the OS values observed in UICC stage II/stage III colon cancer patients receiving adjuvant 5-FU chemotherapy (Ribic *et al*, 2003). In that study, MSI-H colon cancer patients who were not treated with chemotherapy showed an OS of more than 80%, which tended to be better than in the treated group. Our data thus support previous publications, suggesting that adjuvant chemotherapy might not be beneficial in MSI-H colon cancer patients of UICC stage II and potentially also stage III.

Mutations of the *B2M* gene have been reported as a frequent event in MSI-H colon cancer (Bicknell *et al*, 1996; Kloor *et al*, 2005). The absence of liver metastases in patients with *B2M*-mutant MSI-H colon cancer led us to hypothesise that *B2M* mutations might interfere with metastasis formation and thus have a potentially favourable prognostic effect in MSI-H colon cancer patients (Kloor *et al*, 2007). Thus, subgroup analysis was performed to determine a potential prognostic role of *B2M* mutation status within the MSI-H colon cancer group.

*Beta2-Microglobulin* mutations were detected in 10 (29.4%) out of 34 MSI-H colon cancers. This frequency is in the range of our previous study (Kloor *et al*, 2007). No relapse events and no death events related to the primary tumour were registered in the group of patients with *B2M*-mutant MSI-H colon cancer. Subclassification of MSI-H colon cancer patients according to *B2M* mutation status revealed that *B2M* wild-type MSI-H colon cancer patients were comparable to MSS colon cancer patients with regard to TTR. This is suggestive of the hypothesis that the improved prognosis previously reported for MSI-H colon cancer patients is related to the frequent occurrence of *B2M* mutations in this tumour type. Moreover, one might speculate that *B2M* mutation status is a more accurate prognostic marker than MSI status alone.

The mechanism contributing to a decreased metastatic potential of *B2M*-mutant, HLA class I-deficient colon cancer cells may involve NK cell-mediated tumour cell lysis (Jager *et al*, 2002). This hypothesis is supported by the observation that, MHC class I-deficient uveal melanomas were highly sensitive to NK cell-mediated killing and did not develop liver metastases in a murine model (Ma *et al*, 1995). In line with these observations, patients with HLA class I antigen-deficient uveal melanoma had a better prognosis compared with their HLA class I antigen-proficient counterparts (Blom *et al*, 1997; Jager *et al*, 2002). Alternatively, *B2M* mutations may directly influence the oncogenic potential of MSI-H colon cancer cells in an HLA-independent manner, as *B2M* has recently been demonstrated to enhance epithelial-to-mesenchymal transition and promote bone metastasis in several human cancer types (Huang *et al*, 2006; Josson *et al*, 2011). Further studies on the mechanisms underlying *B2M* inactivation and metastasis formation in MSI-H colon cancer will be warranted.

In summary, our results indicate that MSI status modulates the risk of disease relapse in colon cancer patients in a time-varying manner, with a significantly reduced risk of disease relapse in MSI-H compared with MSS colon cancer patients after 12 months of follow-up. Moreover, *B2M* mutations were associated with the absence of metastasis formation and disease relapse. This suggests that *B2M* mutations, beyond their association with M0 stage at the time point of colon cancer diagnosis, may predict a favourable outcome in MSI-H colon cancer patients. *B2M* mutation analysis should therefore be included as a potential prognostic marker in future colon cancer therapy trials.

## Figures and Tables

**Table 1:** Clinical characteristics of the sample population of 269 FOGT4 trial patients with UICC stage II or stage III colon cancer, according to microsatellite instability status

					**MSI-H**	
**Characteristic**	**FOGT-4 trial (*N*=269)**	**Evaluable for MSI (*N*=223)**	**MSS (*N*=189)**	**MSI-H (*N*=34)**	***B2M* WT (*N*=24)**	***B2M* mutation** **(*N*=10)**
Median age (years)	64	64	64	66	63	69
Male gender	157 (58%)	133 (60%)	116 (61%)	17 (50%)	12 (50%)	5 (50%)
						
*T-stage*						
2	24 (9%)	20 (9%)	18 (10%)	2 (6%)	1 (4%)	1 (10%)
3	174 (65%)	141 (63%)	122 (65%)	19 (56%)	14 (58%)	5 (50%)
4	71 (26%)	62 (28%)	49 (26%)	13 (38%)	9 (38%)	4 (40%)
						
*N-stage*						
0	40 (15%)	32 (14%)	24 (13%)	8 (24%)	4 (17%)	4 (40%)
1	149 (55%)	128 (57%)	112 (59%)	16 (47%)	11 (46%)	5 (50%)
2	78 (29%)	61 (27%)	51 (27%)	10 (29%)	9 (38%)	1 (10%)
x	2 (1%)	2 (1%)	2 (1%)	—	—	—
						
*Chemotherapy*						
5FU/FA + CPT-11	136 (51%)	113 (51%)	99 (52%)	14 (41%)	10 (42%)	4 (40%)
5FU/FA	133 (49%)	110 (49%)	90 (48%)	14 (58%)	6 (60%)	90 (48%)

Abbreviations: B2M=Beta2-Microglobulin; FA=folinic acid; 5FU=5-fluorouracil; MSI-H=high-level microsatellite instability; MSS=microsatellite stable; UICC=Union for International Cancer Control; WT=wild type.

**Table 2:** Cox regression model with time-varying effect of MSI status

**Time-varying effects**		
	**Test for time invariant effects**	**Test for nonsignificant effects**
MSI status	*P*=0.002	*P*<0.001
		

**Time-constant effects**			
	**HR**	**95% CI**	***P*-value**
Age (years)	0.99	0.97–1.02	0.70
Gender (male *vs* female)	0.98	0.48–1.49	0.95
Treatment (5FU/FA *vs* 5FU/FA+CPT-11)	0.87	0.35–1.39	0.59
T-stage (4 *vs* 2,3)	2.25	1.67–2.84	0.007
N-stage (1 *vs* 0)	3.22	2.17–4.27	0.03
(2 *vs* 0)	4.81	3.69–5.92	0.006

Abbreviations: CI=confidence interval; FA=folinic acid; 5FU=5-fluorouracil; HR=Hazard ratio; MSI=microsatellite instability.
